# Proteomic profiling reveals the molecular signatures of chemotherapy-induced human ovarian damage

**DOI:** 10.1093/humrep/deaf203

**Published:** 2025-11-05

**Authors:** Johanne Grosbois, Xavier Bisteau, Virginie Imbault, Louise Conrard, Margherita Condorelli, Necati Findikli, Pascale Lybaert, Isabelle Demeestere

**Affiliations:** Research Laboratory on Human Reproduction, Université Libre de Bruxelles (ULB), Brussels, Belgium; Institute of Interdisciplinary Research (IRIBHM) J.E.Dumont & Analytical Platform of the Faculty of Pharmacy (APFP), Université Libre de Bruxelles (ULB), Brussels, Belgium; Institute of Interdisciplinary Research (IRIBHM) J.E.Dumont & Analytical Platform of the Faculty of Pharmacy (APFP), Université Libre de Bruxelles (ULB), Brussels, Belgium; Center for Microscopy and Molecular Imaging (CMMI), Université Libre de Bruxelles, Gosselies, Belgium; Department of Obstetrics and Gynecology, Fertility Clinic, HUB Erasme, Brussels, Belgium; Department of Obstetrics and Gynecology, Fertility Clinic, HUB Erasme, Brussels, Belgium; Research Laboratory on Human Reproduction, Université Libre de Bruxelles (ULB), Brussels, Belgium; Research Laboratory on Human Reproduction, Université Libre de Bruxelles (ULB), Brussels, Belgium; Department of Obstetrics and Gynecology, Fertility Clinic, HUB Erasme, Brussels, Belgium

**Keywords:** ovarian tissue, fertility preservation, chemotherapy, ovarian reserve, stromal cells, ovarian microenvironment, alkylating agents, cryopreservation

## Abstract

**STUDY QUESTION:**

How does first-line chemotherapy alter follicular survival and the ovarian microenvironment?

**SUMMARY ANSWER:**

First-line chemotherapy exposure prior to ovarian tissue cryopreservation (OTC) induces follicular DNA damage and apoptosis, and causes microenvironmental alterations including immune dysfunction, increased hypoxia and apoptosis, impaired cell cycle and DNA repair capacity, and disruption of the extracellular matrix (ECM).

**WHAT IS KNOWN ALREADY:**

Although the mechanisms underlying chemotherapy-induced damage to germ cells are being increasingly deciphered, its impact on the ovarian microenvironment remains largely unexplored. The ovarian stroma is equally exposed to chemotherapy, and since its cells and components are in active communication with follicles, any microenvironmental changes induced by chemotherapy might affect follicles as well.

**STUDY DESIGN, SIZE, DURATION:**

Cryopreserved ovarian cortex samples from 10 cancer patients (aged 24–30 years) who donated tissue for research purposes were analyzed. Of the 10 patients, 5 had received first-regimen chemotherapy and 5 age-matched controls had not undergone chemotherapy prior to OTC. Chemotherapy-induced ovarian injury was evaluated by comparing stromal and follicular alterations between chemotherapy-exposed and control patients.

**PARTICIPANTS/MATERIALS, SETTING, METHODS:**

Cryopreserved ovarian cortex were processed directly after thawing. Proteins and biological processes dysregulated in response to chemotherapy were identified by mass spectrometry, and the data obtained were validated by western blotting and immunohistochemistry. Stromal and follicular alterations were further examined by (immuno)histochemistry, focusing on apoptosis (TUNEL), DNA damage (γH2Ax), oxidative stress (8-OHdG), proliferation (Ki67), fibrosis (picrosirius red), and analysis of follicle number, developmental stage and morphology.

**MAIN RESULTS AND THE ROLE OF CHANCE:**

A total of 5209 proteins were detected in both chemotherapy-exposed and control ovaries, of which 237 proteins (4.5%) showed differential expression. Biological pathways related to immune response, hypoxia and apoptosis were upregulated after chemotherapy exposure, while those involved in cell cycle and DNA repair were downregulated. Markers of the ECM network were also dysregulated. Western blotting and immunostaining confirmed the significant upregulation of complement C3 (innate immunity; *P* = 0.032), SELENBP1 (hypoxia; *P* = 0.030) and KRT18 (apoptosis; *P* = 0.015) as well as a non-significant increase in SERPIN A3 level (ECM; *P* = 0.077) following chemotherapy-exposure, while NCBP2 (DNA repair) was reduced, though not significantly (*P* = 0.067). Targeted analyses demonstrated that despite increased stromal cell density following chemotherapy treatment (1.84 ± 0.15 × 10^6^ cells/mm^3^ vs. 1.62 ± 0.13 × 10^6^ cells/mm^3^; *P* = 0.036), no fibrosis was observed. Stromal apoptosis and oxidative stress levels were comparable in both groups (*P* = 0.464 and *P* = 0.247, respectively). In contrast, first-regimen chemotherapy persistently affected germ cells, increasing follicular apoptosis (*P* = 0.013), DNA damage (*P* = 0.033) and possibly morphological defects (*P* = 0.061), without depleting the ovarian reserve.

**LIMITATIONS, REASONS FOR CAUTION:**

Patients previously exposed to chemotherapy had received different low-gonadotoxic chemotherapy regimens at different times before OTC, making it challenging to isolate the effects of individual agents or to differentiate between short- and long-term dysregulated biological processes. Additionally, analyses were performed on a small cohort and results should be interpreted cautiously.

**WIDER IMPLICATIONS OF THE FINDINGS:**

These findings provide evidence that chemotherapy significantly alters both the ovarian germ cells and stroma, emphasizing the need to further investigate the underlying molecular mechanisms and their impact on ovarian function and fertility preservation. They also suggest target proteins which may drive these chemotherapy-associated ovarian damage for future investigations.

**STUDY FUNDING/COMPETING INTEREST(S):**

This research was funded by a grant from the Fond National de la Recherche Scientifique de Belgique—FNRS (grant 1.B.218.24F awarded to J.G.). The support is provided by the Funds Suzanne Duchesne, Serge Rousseau and Docteur Jean Gérard, managed by the King Baudouin Foundation, and by the Jaumotte-Demoulin Foundation. The CMMI is supported by the European Regional Development Fund and the Walloon Region. J.G. and I.D. are supported by FNRS as a postdoctoral researcher and a senior research associate, respectively. The authors have no conflicts of interest to declare.

**TRIAL REGISTRATION NUMBER:**

N/A

## Introduction

Out of an estimated 19.3 million new cancer cases diagnosed worldwide, 1.9 million occur in girls and women of reproductive age, aged under 50 years (Ferlay *et al.*, 2021). Although survival rates continue to improve for many cancers, chemotherapy-induced off-target ovarian toxicity can dramatically impact survivors’ quality of life, both in terms of reproductive and endocrine outcomes, increasing morbidity and psychological distress (van Dorp *et al.*, 2018; Logan *et al.*, 2019). Girls and young women undergoing chemotherapy are susceptible to gonadal damage and loss of fertility. This can involve partial or complete destruction of ovarian follicles, increasing the risk of premature ovarian insufficiency (POI) and infertility (Overbeek *et al.*, 2017). While oocyte, embryo and ovarian cortex cryopreservation are the current main methods to preserve female fertility, the development of alternatives that protect ovarian function during chemotherapy would represent a pivotal breakthrough (Nguyen *et al.*, 2025). Beyond reproduction, the ovary is a major endocrine organ. Chemotherapy-induced follicle loss can lead to estrogen deficiency increasing the risks of osteoporosis, cardiovascular disease, depression and anxiety (Faubion *et al.*, 2015). Deciphering the mechanisms underlying chemotherapy-induced gonadotoxicity is thus critical and urgently needed, particularly in the context of fertility preservation, and will enable the development of targeted therapies to mitigate off-target toxicity without compromising anticancer efficacy.

The mechanisms driving follicle loss in response to chemotherapy remain not fully elucidated. Existing studies have largely focused on the direct effects of chemotherapy on germ cells, showing that gonadotoxicity is mediated through (i) direct DNA damage in primordial follicles leading to apoptosis; (ii) induction of massive activation and growth of dormant follicles, which are then destroyed; and (iii) vascular injury (reviewed in Spears *et al.*, 2019). However, the ovarian cortex comprises multiple cell populations, including stromal cells (83%), oocytes (0.2%), perivascular cells (10%), endothelial cells (5%), granulosa cells (1.2%), and theca and immune cells (0.4%) (Fan *et al.*, 2019; Wagner *et al.*, 2020). Chemotherapy affects the entire organ, and the pattern of follicle loss is not homogeneous within the ovary (Meirow *et al.*, 2007), suggesting that disruptions in the ovarian microenvironment may also play a critical role in follicular depletion. Given the essential interplay between the ovarian stroma and follicles in regulating ovarian function (Grosbois *et al.*, 2020; Telfer *et al.*, 2023), it is crucial to elucidate the full extent of chemotherapy-induced ovarian injury, including at the stromal level.

Despite limited research in the field, emerging evidence, primarily from rodent models, has revealed that the ovarian microenvironment experiences profound alterations following chemotherapy exposure (detailed in Guo *et al.*, 2024). Among them, stromal fibrosis and destruction of the vascular structure and function have been observed in human ovarian tissue collected post-chemotherapy (Meirow *et al.*, 2007; Pampanini *et al.*, 2019; Shai *et al.*, 2021; Devos *et al.*, 2023). Oxidative stress disturbance, inflammation and alterations in immune cell phenotypes have also been described following gonadotoxic treatment (Huang *et al.*, 2019; Chen *et al.*, 2021; Deng *et al.*, 2021; Du *et al.*, 2022; He *et al.*, 2023).

A few studies have investigated gene expression changes in chemotherapy-exposed human follicles and mouse ovaries and revealed dysregulated apoptotic, metabolic and oxidative stress-related pathways (Li *et al.*, 2019; Titus *et al.*, 2021; Wu *et al.*, 2023; Zhang *et al.*, 2023). However, transcriptional alterations do not always translate into changes in protein levels and biological function. They also do not reflect either the stroma composition or changes that could arise in the extracellular matrix (ECM). By focusing on the final protein products, proteomic approaches offer the advantage of providing a robust and representative picture of the functioning cell and tissue. Previous proteomic studies conducted in human ovaries have identified up to 5253 proteins (Wang *et al.*, 2005; Ouni *et al.*, 2019), establishing a reference map that serves as a valuable resource for comparing physiological and pathological conditions.

The present study used quantitative proteomics to investigate the molecular signatures involved in chemotherapy-induced ovarian damage. By comparing the proteome of ovaries from women who have previously been treated with chemotherapy to ovaries from non-exposed women, we identified key dysregulated ovarian proteins and pathways, laying the groundwork for potential pharmacoprotection strategies to preserve ovarian health. Additional histological and immunological analyses were performed to provide a more comprehensive overview of chemotherapy-induced stromal and follicular damage.

## Materials and methods

### Ethical approval

Human ovarian cortical biopsies were obtained from 10 patients with cancer who underwent ovarian tissue cryopreservation (OTC) for fertility preservation purposes (Table 1). All patients provided written informed consent to donate their residual frozen tissue to research at the end of the storage period. This project was approved by the Erasme Hospital Ethical Committee (Brussels, Belgium) with reference P2023/471.

**Table 1.** Clinical characteristics of the patients included in this study.

Patient	Age at OTC (years)	Cancer diagnosis	Chemotherapeutic regimen	CED (mg/m^2^)	Treatment to OTC interval (months)
Control 1	29	Lymphoma	N/A	N/A	N/A
Control 2	28	Breast cancer	N/A	N/A	N/A
Control 3	30	Lymphoma	N/A	N/A	N/A
Control 4	25	Breast cancer	N/A	N/A	N/A
Control 5	27	Breast cancer	N/A	N/A	N/A
Chemo 1	29	Breast cancer	6 FEC^*^	3000	6
Chemo 2	24	Lymphoma	ABVD	0	< 1
Chemo 3	27	Lymphoma	fludarabine	0	18
Chemo 4	28	Breast cancer	3 FEC^*^	1500	1
Chemo 5	24	Leukemia	ICE	0	2

OTC, ovarian tissue cryopreservation; CED, cumulative cyclophosphamide equivalent dose; FEC, 5-fluorouracil, epirubicin and cyclophosphamide; ABVD, adriamycin, bleomycin, vinblastine and dacarbazine; ICE, idarubicin, carboplatin and etoposide;

* containing alkylating agents.

### Freezing and thawing procedures

The ovarian tissues were cryopreserved using a slow-freezing protocol at Erasme hospital (Brussels, Belgium) and stored in liquid nitrogen. The cortical tissues were rapidly thawed according to the Erasme clinical protocol as previously described (Demeestere *et al.*, 2003, 2006). After thawing, tissue was rinsed in Leibovitz medium (Gibco, Belgium) supplemented with 2 mM sodium pyruvate (Sigma, Belgium), 2 mM glutamine (Sigma), 3 mg/ml human serum albumin (HSA) (CAF DCF, Belgium), 30 μg/ml penicillin G (Sigma) and 50 μg/ml streptomycin (Sigma), then cut into fragments of approximately 4 × 2 mm^2^, with one fragment per patient being randomly selected for mass spectrometry, one fragment for histological analysis and one fragment for western blotting.

### Protein extraction and LC-MS/MS

The 10 ovarian samples used for proteomic analysis (one per patient) were processed in two parallel batches of five samples each for the thawing and protein extraction steps: the first batch included 2 controls and 3 chemotherapy-treated samples, and the second included 3 controls and 2 chemotherapy-treated samples. All 10 samples were then processed simultaneously for the subsequent steps. Ovarian cortical fragments were mechanically ground in 8 M Urea, 50 mM tris-HCl pH 8.5 with an Ultraturrax (IKA Werke GmbH & Co. KG, Germany), homogenized using Lysing Matrix D beads in a TissueLyzer II (Qiagen, Germany) at 30 Hz for 10 min and sonicated until clear. Lysates were centrifuged at 16 000 g for 10 min, and protein concentrations in the supernatants were determined by paper method (Minamide and Bamburg, 1990). Fifty micrograms of proteins from each sample were reduced with 10 mM Tris(2-carboxyethyl)phosphine (TCEP) (Merck, Germany) for 30 min at room temperature and subsequently alkylated with 55 mM chloroacetamide (Merck) for 30 min at room temperature in the dark. The solution was then diluted eight times with 100 mM triethylammonium bicarbonate (TEAB) (Merck) and proteins were digested overnight with 1 µg of trypsin (Promega, WI, USA) and 1 µg of LysC (Wako, VA, USA) at 37 °C under agitation. Peptides were retained on HLB SPE columns (Spin15- HLB.T3.50; Affinisep, France) and eluted with Acetonitrile (ACN):H_2_O (65:35 v/v) 0.1% v/v Formic acid (FA). After evaporation, samples were reconstituted in H_2_O with 0.1% FA before injection.

Peptides were separated on a separation column (ChromXP C18 CL, 150 × 0.3 mm, 3 µm; Sciex, MA, USA) using a two-step ACN gradient (5–25% ACN/0.1% FA in 65 min then 25–60% ACN/0.1% FA in 25 min) and sprayed online in a TTOF5600 (Sciex) mass spectrometer. SWATH/DIA acquisitions were performed using 71 windows of variable effective isolation width to cover a mass range of 400-1250 m/z. SWATH MS2 spectra were collected from 50 to 2000 m/z. The collision energy for each window was determined according to the calculation for a charge 2+ ion centered upon the window with a spread of 15. An accumulation time of 45 ms was used for all fragment-ion scans in high-sensitivity mode and for the survey scans in high-resolution mode acquired at the beginning of each cycle, resulting in a duty cycle of ∼3.3 s.

### Mass spectrometry data analysis

Raw data files were converted to mzML format with the MSConvert program (v.3.0). The mzML files were carried out with DIA-NN (v.1.8.2 beta 27) (Demichev *et al.*, 2020) with search parameters set as follows: precursor FDR 1%; mass accuracy at MS1 and MS2 both set to 0; scan window set to 0; isotopologues and MBR turned on; protein inference at gene level; heuristic protein inference enabled; quantification strategy set to QuantUMS (high accuracy); neural network classifier double-pass mode; and cross-run normalization RT-dependent. A universal library was used, and protein re-annotation was performed. Spectra were searched against a prior *in loco* generated library containing 9972 proteins covered by 174347 peptides including common contaminants.

To generate the library, DDA and DIA data previously acquired on TT5600 were used to generate one universal library by FragPipe (v.18.0) using a pre-defined workflow DIA_SpecLib_Quant. Specifically, decoys were first added to the FASTA which contains human protein sequences (UP000005640, 83374 reviewed and unreviewed entries, downloaded from UniProtKB on April 7, 2023). Then, MSFragger (v.3.5) was used to search DDA raw data (Kong *et al.*, 2017) with the following settings: precursor and fragment mass tolerance 30 ppm; strict trypsin with no more than five missed cleavages; peptide length 6–50; peptide mass 500–5000; C + 57.021464 as fixed modification; M + 15.9949, N-term +42.0106, nQ -17.0265, nE -18.0106, DN +0.984016 and carbamylation +43.005814 as variable modifications; min matched fragments 4; and max fragment charge 2. In the validation step, MSBooster was implemented on both spectra and RT levels (Yang *et al.*, 2023), and then Percolator and ProteinProphet integrated in Philosopher (v.4.4.0) were used for PSM validation and protein inference (da Veiga Leprevost *et al.*, 2020). Library generation was conducted using EasyPQP (IonQuant v.1.9.8) with RT calibration based on ciRTs and Lowess fraction set to 0.04. Only fragment types b and y were included with a tolerance of 15 ppm with a max delta_unimod.

The protein group output matrix and associated experiment annotation files were imported into FragPipe-Analyst interface for differential abundance testing (Hsiao *et al.*, 2024). The minimum percentage of non-missing values was set to 60% in at least one condition. A cutoff of the adjusted value of *P* < 0.05 (Benjamini-Hochberg method) along with an absolute log2 fold change of 0.585 was applied to determine significantly dysregulated proteins in each pairwise comparison.

### Western blotting

Ovarian fragments were chopped and lysed in Laemmli buffer containing proteases and phosphatases inhibitors (Sigma) using a MagNA Lyser instrument (Roche) as previously described (Grosbois and Demeestere, 2018). The protein samples were denatured by boiling for 5 min, separated on a 5–12% polyacrylamide gel, and electrophoretically transferred onto nitrocellulose membranes (Bio-Rad, Belgium). Membranes were blocked then probed with antibodies against complement C3 (21337-1-AP, Proteintech, 1:50 000), KRT18 (10830-1-AP, Proteintech, 1:500), p27 (3686, Cell Signaling, 1:1000), SELENBP1 (27230-1-AP, Proteintech, 1:5000), SERPIN A3 (12192-1-AP, Proteintech, 1:10000) or ACTIN (4967, Cell Signaling, 1:500) overnight at 4 °C, then incubated with a horseradish peroxidase conjugated secondary antibody (7074, Cell Signaling, 1:1000). Protein bands were detected using the SuperSignal West Femto Chemiluminescent Substrate (Thermo Scientific, Belgium), imaged using a Fusion FX imager (Vilber, France) and quantified using Image Lab software. Target proteins expression was normalized to ACTIN protein levels (internal loading control).

### Histology

After fixation in 4% paraformaldehyde for 24 h, ovarian fragments were dehydrated in increasing concentrations of ethanol (70–100%), embedded in paraffin and serially sectioned at 5 μm thickness. Every 10th section was stained with hematoxylin and eosin to evaluate follicular developmental stage, density and morphological evaluation as well as stromal cell density; the remaining sections were used for histological and immunohistological analysis.

Follicles were classified according to their developmental stage as previously described (Yano Maher *et al.*, 2025): primordial follicles (oocyte surrounded by a few flattened granulosa cells—GCs), transitional follicles (oocyte surrounded by flattened and at least one cuboidal GC) or growing follicles (oocyte surrounded by one or more complete layer(s) of cuboidal GCs). Each follicle with a visible nucleus was counted and staged as morphologically normal or abnormal based on criteria previously specified (Yano Maher *et al.*, 2025). The presence of multi-oocyte and multi-nucleated follicles was also reported. Follicle density was calculated by dividing the total number of follicles per patient by the volume of paraffin-embedded tissue analyzed. Stromal cell nuclei were counted using a custom FIJI macro (Image J), and stromal cell density was calculated by dividing the number of stromal cells by the volume of paraffin-embedded tissue analyzed (N = 5 patients/group; N = 5 sections per patient).

To investigate the density and packing of collagen I and III fibers, the PicroSirius Red (PSR) staining (ab246832, Abcam, UK) was performed. Briefly, deparaffinized and rehydrated slides were immersed in a PSR staining solution for 1 h at room temperature, then washed twice with 0.5% glacial acetic acid before dehydration and mounting.

### Immunohistology

Immunohistochemistry and immunofluorescence were performed for the validation of mass-spectrometry-identified chemotherapy-targeted proteins (SERPIN G1, NCBP2) and the assessment of oxidative stress (8-OHdG), cell proliferation (Ki67) and DNA damage (γH2Ax). Antigen retrieval by heat was performed in citrate buffer pH 6.0 (8-OHdG, Ki67, γH2Ax) or EDTA buffer pH 8.5 (SERPIN G1, NCBP2), and endogenous peroxidase activity was quenched using 3% (v/v) hydrogen peroxide (Carl Roth, Belgium). Non-specific binding sites were blocked using 5% v/v normal goat serum then the slides were incubated with SERPIN G1 (12259-1-AP, Proteintech, 1:1000), NCBP2 (11950-1-AP, Proteintech, 1:3000), 8-OHdG (SAB5200010, Merck, 1:500), Ki67 (556003, BD Biosciences, 1:400) or γH2Ax (05-636, Merck, 1:100) antibodies overnight at 4 °C. For SERPIN G1, NCBP2, 8-OHdG and Ki67, sections were incubated with a biotinylated secondary antibody (Vector Laboratories, UK) and processed using an ABC kit according to the manufacturer’s instructions (Vectastain Elite ABC kit; Vector Laboratories). The signal was visualized using 3,3′-diaminobenzidine (DAB) peroxidase substrate kit (Vector) then counterstained with hematoxylin. For γH2Ax, the slides were incubated with a biotinylated goat anti-mouse secondary antibody then with avidin-Texas Red (1:200, Vector) and counterstained with Hoechst. Negative controls were obtained by replacing the primary antibody with blocking solution containing non-immune serum. All washes were performed in PBS or PBS with 0.05% (v/v) Triton at room temperature.

### Apoptosis assay

DNA fragmentation was assessed by TdT-mediated dUTP-biotin nick-end labeling (TUNEL) using the In Situ Cell Death Detection Kit (Roche, Germany). Deparaffined and rehydrated sections were permeabilized with 20 μg/ml proteinase K in 10 mM Tris pH7.4 (Qiagen, Netherlands). After washing, the slides were labeled with the kit reagents according to the manufacturer instructions and counterstained with Hoechst (1 μg/ml). Positive and negative controls were obtained by treatment with 50 IU/ml DNAse I (Invitrogen, USA) and enzyme-free buffer solution, respectively.

### Image analysis and quantification

Whole ovarian sections were scanned using a Nanozoomer-SQ scanner (Hamamatsu Photonics, Belgium—brightfield images, ×40 magnification) and an Axioscan 7 (Zeiss, Germany—fluorescent images using a Plan-Apochromat 20×/0.8 objective, and birefringent images using an EC Plan-Neofluar 20×/0.5 objective). The percentage of SERPIN G1-, TUNEL-, 8-OHdG- and PSR-positive area was calculated in five sections per patient distanced at least 100 µm apart, using the threshold tool in FIJI (N = 5 patients/experimental group; N = 5 sections per patient). NCBP2-positive nuclei were counted using a custom FIJI macro and reported as a percentage of the total number of stromal cell per section (N = 5 patients/experimental group; N = 5 sections per patient).

PSR samples were further analyzed under polarized light to assess collagen fiber diameter and packing density. Collagen fiber color was quantified using a custom FIJI macro as previously described (Grosbois *et al.*, 2023). Briefly, the hue (color) of each pixel was determined and a color threshold was used to isolate the three main colors seen in PSR-stained samples under polarized light: red (thick fibers), yellow (mid-sized fibers) and green (thin fibers). The thresholds on the hue histogram were set as follows: red 2–9, yellow/orange 10–38 and green 24–135. The relative percentage of each color was calculated by dividing the pixel count of each color by the total pixel count for each image.

Ki67- and 8-OHdG-positive follicles (one or more positive GCs) as well as γH2AX- and TUNEL-positive follicles (positive oocyte, one or more positive GCs or both cell types) were counted and reported as a percentage of the total number of follicles per developmental stage and per patient.

### Statistical analysis

Statistical analyses related to mass spectrometry are described in the Mass spectrometry data analysis section. For experiments involving immunoblot, histology and immunostaining quantification, statistical analyses were performed using SPSS version 29. Homogeneity of variance was verified using Levene’s test, and unpaired two-tailed t-tests were used to determine significant differences between the two experimental groups. A *P*-value < 0.05 was considered statistically significant. Data are presented as mean ± SD or as scatter plots ± SD.

## Results

### Population characteristics

A total of 10 patients were included in this study. Among them, five had received first-line chemotherapy before OTC and five had not. The mean age of control and chemotherapy-exposed patients was 27.8 ± 1.9 and 26.4 ± 2.3 years, respectively (mean ± SD; *P* = 0.327). Patient age, diagnosis, chemotherapeutic regimen, cumulative cyclophosphamide equivalent dose (CED) (Green *et al.*, 2014) and time interval between treatment and OTC are detailed in Table 1.

### Chemotherapy-exposed and control ovaries have distinct proteomic signatures

**Figure 1.**
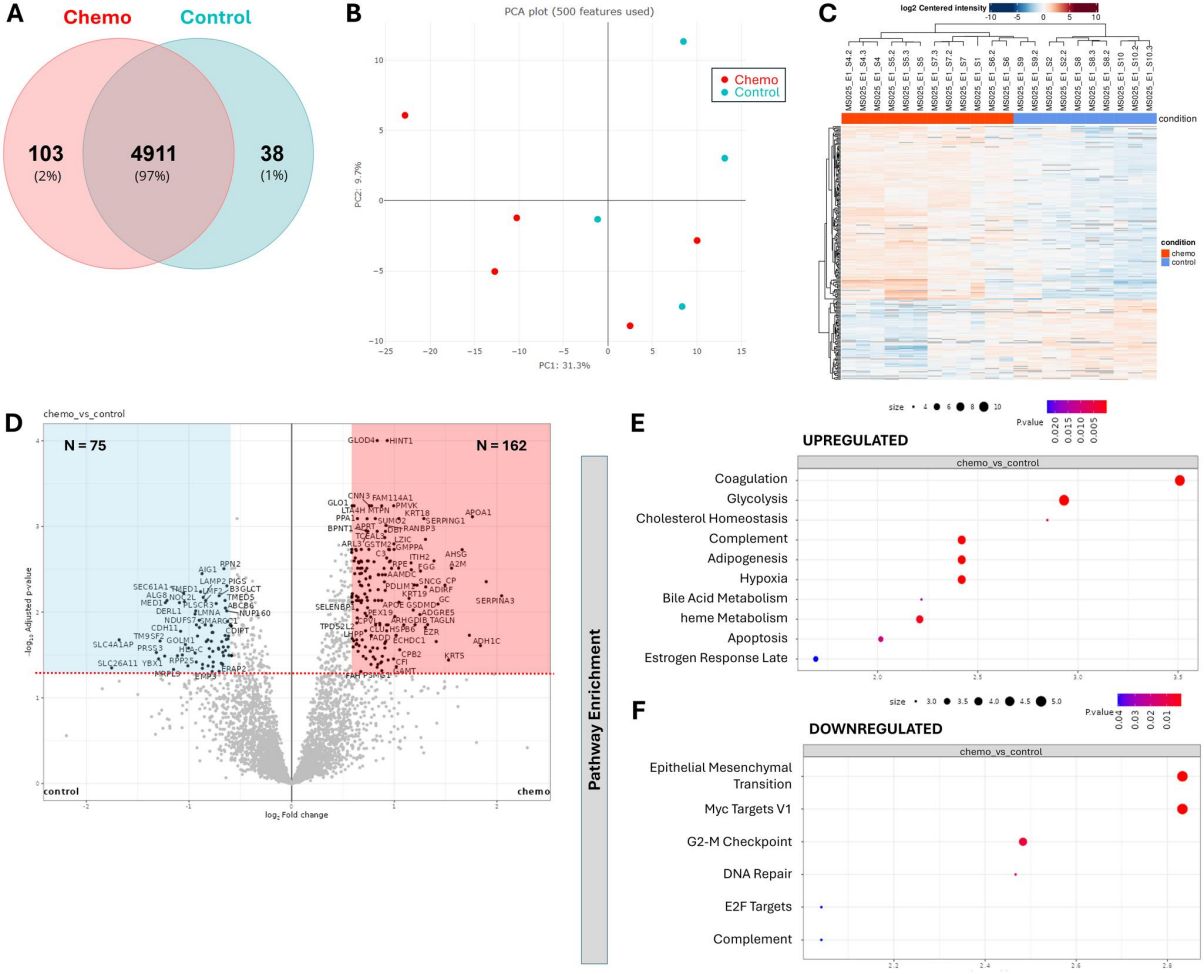
**Comparative mass-spectrometry analysis of cryopreserved-thawed ovarian cortex collected from women exposed or not to first-line chemotherapy before ovarian tissue cryopreservation.** (**A**) Venn diagram showing the overlapping proteins between the two experimental groups. (**B**) Principal component analysis (PCA) plot showing the similarities between the samples. (**C**) A heatmap of differential abundance of proteins in each of the replicates; rows represent the differential abundance of proteins, and columns represent the sample replicates. (**D**) Volcano plot showing the upregulated (N = 162, red) and downregulated (N = 75, blue) proteins in chemotherapy-exposed ovaries compared to untreated ovaries (the dashed red line indicates adjusted *P* < 0.05). (**E**, **F**) Top 10 significantly upregulated (E) and downregulated (F) enriched pathways from hallmark gene sets. Only proteins with log2 fold change > 0.585 and *P*-value < 0.05 were considered. For all significant terms the corrected *P*-value, group size and the enrichment factor are displayed. N = 4 in control group, N = 5 in chemotherapy group.

To study the overall protein expression changes in response to chemotherapy, we conducted a proteomic profiling to compare protein abundance in ovarian cortex pieces from patients exposed to chemotherapy or not prior OTC. After quality control, 9 samples out of 10 were eligible for analysis. One sample in the control group was excluded as it did not meet the set quality standards. A total of 5209 proteins were detected with a false discovery rate (FDR) of 1%. Among them, 4911 were shared between the two groups and retained to perform further differential expression analysis (Fig. 1A). Principal component analysis (PCA) demonstrated partial clustering of the ovarian proteome from chemotherapy-treated and control patients, indicating distinct treatment-dependent proteomic signatures (Fig. 1B). Interestingly, the main differences were observed in tissue from patients with the shorter interval between treatment and OTC (≤ 2 months before OTC). Comparative proteomic analysis revealed 237 differentially expressed proteins (DEPs) (log2 fold change > 0.585; adjusted *P*-value < 0.05), among which 162 were upregulated and 75 were downregulated in the ovaries of chemotherapy-treated patients compared to controls (Fig. 1C and D; Supplementary Tables S1 and S2).

In order to identify the biological processes impacted by chemotherapy, pathway enrichment analysis was conducted with the Molecular Signatures Database (MSigDB) using the Hallmark gene sets as reference. Proteins enriched in pathways involved in the immune response (Coagulation—*P* = 1.6 × 10^−8^; Complement—*P* = 2.2 × 10^−4^), hypoxia (*P* = 2.2 × 10^−4^) and apoptosis (*P* = 9.9 × 10^−3^) were upregulated following chemotherapy exposure, whereas those involved in the ECM remodeling (Epithelial Mesenchymal Transition—*P* = 9.9 × 10^−4^), cell cycle (G2-M Checkpoint—*P* = 7.1 × 10^−3^) and DNA repair (*P* = 0.02) were downregulated (Fig. 1E and F). Similarly, gene ontology (GO) analysis of DEPs revealed that GO terms associated with the immune system response and inflammation, including “regulation of complement activation” (GO : 0030449), “regulation of immune effector process” (GO : 0002697), “regulation of humoral immune response” (GO : 0002920), “neutrophil degranulation” (GO : 0043312), “neutrophil activation involved in immune response” (GO : 0002283) and “neutrophil mediated immunity” (GO : 0002446), were among the top 10 biological processes upregulated following chemotherapy (Supplementary Fig. S1). The GO term “collagen-containing extracellular matrix” (GO : 0062023) was also upregulated after chemotherapy exposure (Supplementary Fig. S1). These results show that proteins involved in immune function, hypoxia, apoptosis, cell cycle, DNA repair capacity and ECM remodeling are significantly dysregulated following chemotherapy treatment.

### Validation of selected differentially expressed proteins

**Figure 2.**
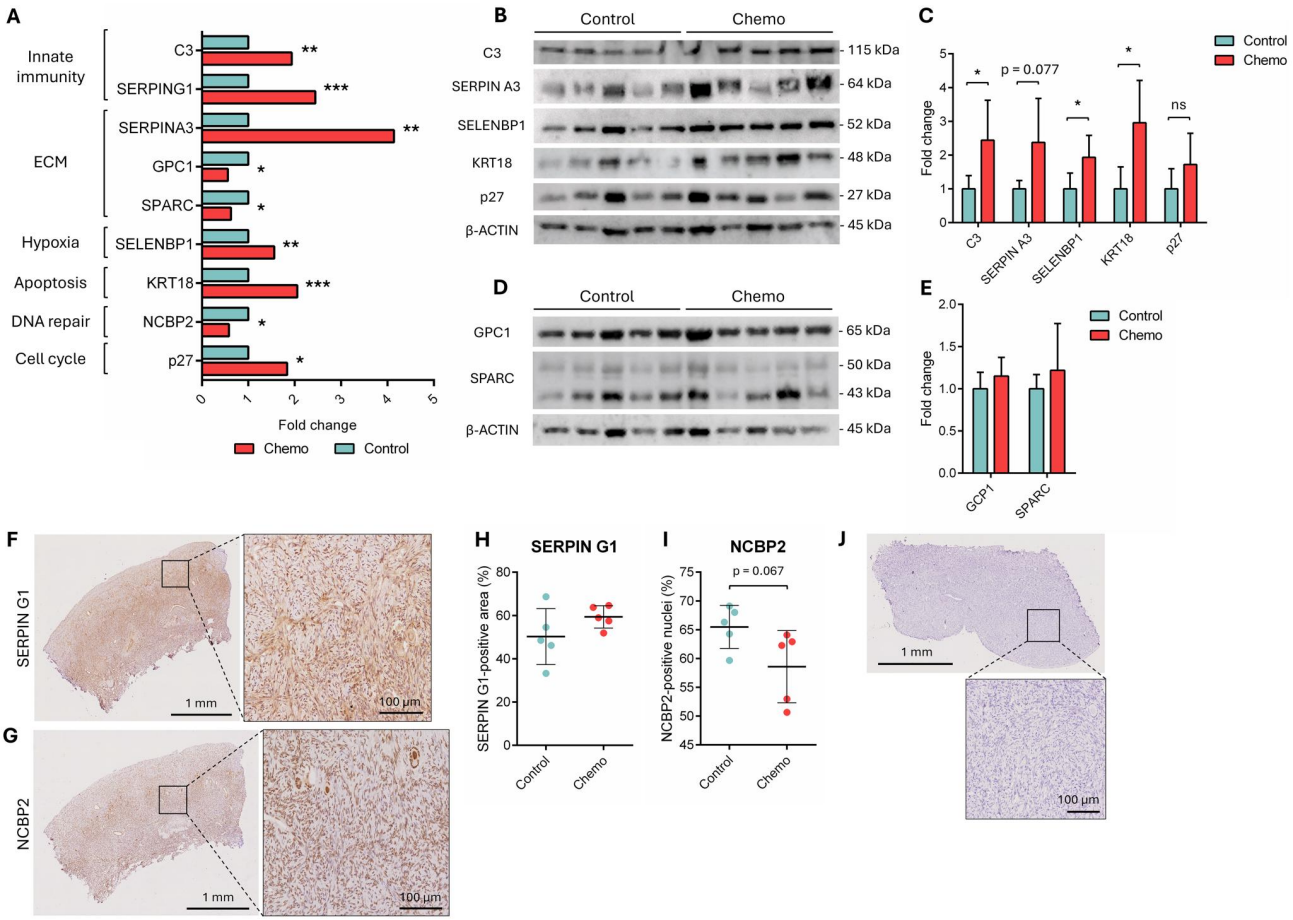
**Validation of the proteomic results by western blotting and immunohistochemistry.** (**A**) Differential expression of selected proteins as detected by mass spectrometry. (**B**, **C**) Expression of upregulated proteins by western blot (B) and quantification of protein levels normalized to ACTIN content (C). (**D**, **E**) Expression of downregulated proteins by western blot (D) and quantification of protein levels normalized to ACTIN content (E). (**F**, **G**) Representative images of human ovarian cortical sections stained with SERPIN G1 (F) and NCBP2 (G). (**H**, **I**) Quantification of the percentage of SERPIN G1-positive area and NCBP2-positive nuclei. (**J**) Negative control for the immunostaining. Data are expressed as mean ± SD or scatter plots ± SD; N = 5 per experimental group. * *P* < 0.05; ** *P* < 0.01; *** *P* < 0.001; ns: not significant. ECM, extracellular matrix.

Validation of the differential abundance of selected DEPs was carried out by western blotting and immunohistology. These DEPs were chosen based on their involvement in the dysregulated biological processes described above (Fig. 2A). Western blotting confirmed the significant upregulation of complement C3 (innate immunity; *P* = 0.032), SELENBP1 (hypoxia; *P* = 0.030) and KRT18 (apoptosis; *P* = 0.015) as well as a non-significant increase in SERPIN A3 level (ECM; *P* = 0.077) following chemotherapy exposure, while p27 (cell cycle) remained stable (*P* = 0.182) (Fig. 2B and C). ECM-related proteins GPC1 and SPARC, predicted to be downregulated post-chemotherapy, displayed no significant difference in expression between groups (*P* = 0.291 and *P* = 0.427, respectively) (Fig. 2C and D). Immunostaining confirmed non-significant higher percentage of SERPIN G1-positive area (innate immunity; 59.4% ± 5.2% vs. 50.3% ± 12.9%, *P* = 0.181) and lower percentage of NCBP2-positive nuclei (DNA repair; 58.6% ± 6.3% vs. 65.5% ± 3.7%, *P* = 0.067) within the ovaries of chemotherapy-treated women compared to controls (Fig. 2D–H). These data largely support the proteomic profiling results, confirming the differential expression of multiple, though not all, candidate proteins.

### Chemotherapy causes cell apoptosis and DNA damage, particularly in the germ cell compartment, but does not induce oxidative stress

**Figure 3.**
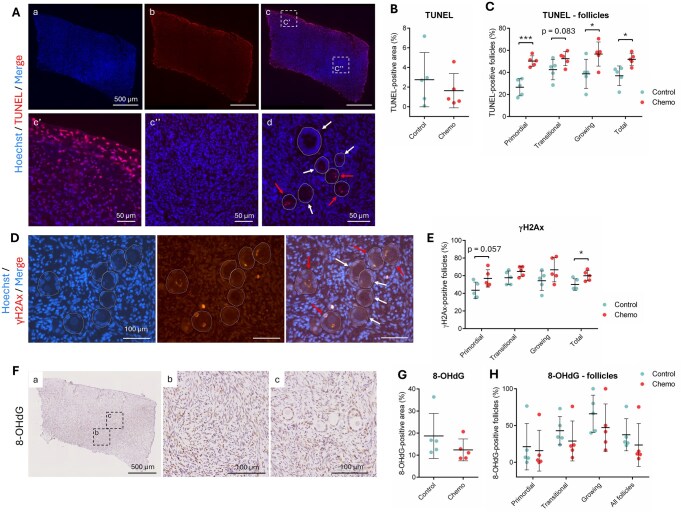
**Impact of chemotherapy on cell apoptosis, DNA damage and oxidative stress.** (**A**) Representative image of a human ovarian section stained with Hoechst (blue, a), TUNEL (red, b) and the merged image (c). (c’) and (c’’) show higher magnifications of insets in (c). (d) shows a photomicrograph of follicles displaying a positive- (red arrows) or negative- (white arrows) TUNEL staining. Scale bar a—c = 500 µm; c’,c”,d = 50 µm. (**B**, **C**). Quantification of the percentage of TUNEL-positive area in the stroma (B) and in follicles (C). (**D**) γH2Ax staining showing positively (red arrows) and negatively (white arrows) stained follicles. Scale bar = 100 µm. (**E**). Quantification of the percentage of γH2Ax-positive follicles. (**F**) Representative image of a human ovarian section stained with 8-OHdG (a). (b) and (c) show higher magnifications of insets in (a) illustrating staining in the stroma and in follicles, respectively. Scale bar a = 500 µm; b, c = 100 µm. (**G**, **H**) Quantification of the percentage of 8-OHdG-positive area in the stroma (G) and in follicles (H). Data presented as scatter plots ± SD; N = 5 per experimental group. * *P* < 0.05; *** *P* < 0.001.

Key biological processes were further investigated, including ovarian cell apoptosis, DNA damage and oxidative stress. The localization and proportion of apoptotic cells is shown in Fig. 3A–C. In the stroma, TUNEL staining was predominantly localized at the edges of the sections while signal was absent in the center. No profound apoptosis was detected (<3%), with no difference between the two experimental groups (*P* = 0.464). At the follicular level, women previously exposed to chemotherapy displayed higher rates of TUNEL-positive follicles compared to controls, significant in primordial, growing and total follicles (*P* < 0.001, *P* = 0.048 and *P* = 0.013, respectively; N = 1162 follicles from 10 patients analyzed; Fig. 3C). Given that ovarian tissue was harvested from women treated with alkylating agents (AA) or non-AA containing chemotherapy from < 1 month up to 18 months before OTC, we further compared the impact of chemotherapy according to the regimens [chemotherapy containing AA (N = 2) vs. chemotherapy without AA (N = 3)] and the time interval between treatment and OTC [≤ 2 months (N = 3) vs. > 2 months (N = 2)] on tissue integrity and follicle survival (Table 1; Supplementary Fig. S2). TUNEL staining remained similar in ovarian cortex whatever the treatment or time interval before OTC (Supplementary Fig. S2A and C). The highest rate of TUNEL-positive follicles was observed in the chemotherapy with AA subgroup, at all developmental stages (Supplementary Fig. S2G). In contrast, the time interval between the last chemotherapy exposure and OTC did not seem to influence follicle survival (Supplementary Fig. S2H).

We also detected phosphorylated histone H2AX at serine 139 (γH2AX) as a marker of DNA double-strand breaks to assess DNA damage response (Fig. 3D). Exposure to chemotherapy significantly increased the percentage of γH2AX-positive follicles compared to controls, rising from 50.0% ± 6.5% to 59.9% ± 5.7% (total follicles, *P* = 0.033; N = 1160 follicles from 10 patients analyzed; Fig. 3E). Primordial follicles appeared to be more sensitive to chemotherapy-induced DNA damage than growing follicles (primordial follicles, *P* = 0.057). Moreover, primordial follicles exposed to chemotherapy with AA displayed higher rates of γH2AX-positive staining compared to those from the control and the chemotherapy without AA subgroups (Supplementary Fig. S2I). Time interval between chemotherapy and OTC did not seem to be correlated with follicular γH2AX staining (Supplementary Fig. S2J).

Given that excessive reactive oxygen species (ROS) production can lead to ovarian dysfunction and diseases (Liang *et al.*, 2023), we measured the level of oxidative stress using 8-OHdG as a marker of oxidative damage to DNA (Fig. 3F). Quantification of 8-OhdG-positive area revealed that oxidative stress damage was similar in the two experimental groups, both in the stroma (*P* = 0.247; Fig. 3G) and in follicles (primordial follicles, *P* = 0.782; transitional follicles, *P* = 0.375; growing follicles, *P* = 0.337; total follicles, *P* = 0.418; N = 834 follicles from 10 patients analyzed; Fig. 3H). It is important to note the presence of one outlier result in each group, with consistently high 8-OHdG level both in the stroma and in follicles. The outlier from the chemotherapy group was ovarian tissue from a patient diagnosed with leukemia, a cancer characterized by increased ROS levels and high oxidative stress status (Trombetti *et al.*, 2021); in contrast, the outlier from the control group was ovarian tissue from a patient diagnosed with lymphoma, not known to induce particular ROS. Last, assessment of stromal cell proliferation using Ki-67 demonstrated rare to no Ki-67 staining in the stroma in all the ovarian cortex assessed.

### First-line chemotherapy is not associated with ovarian fibrosis

**Figure 4.**
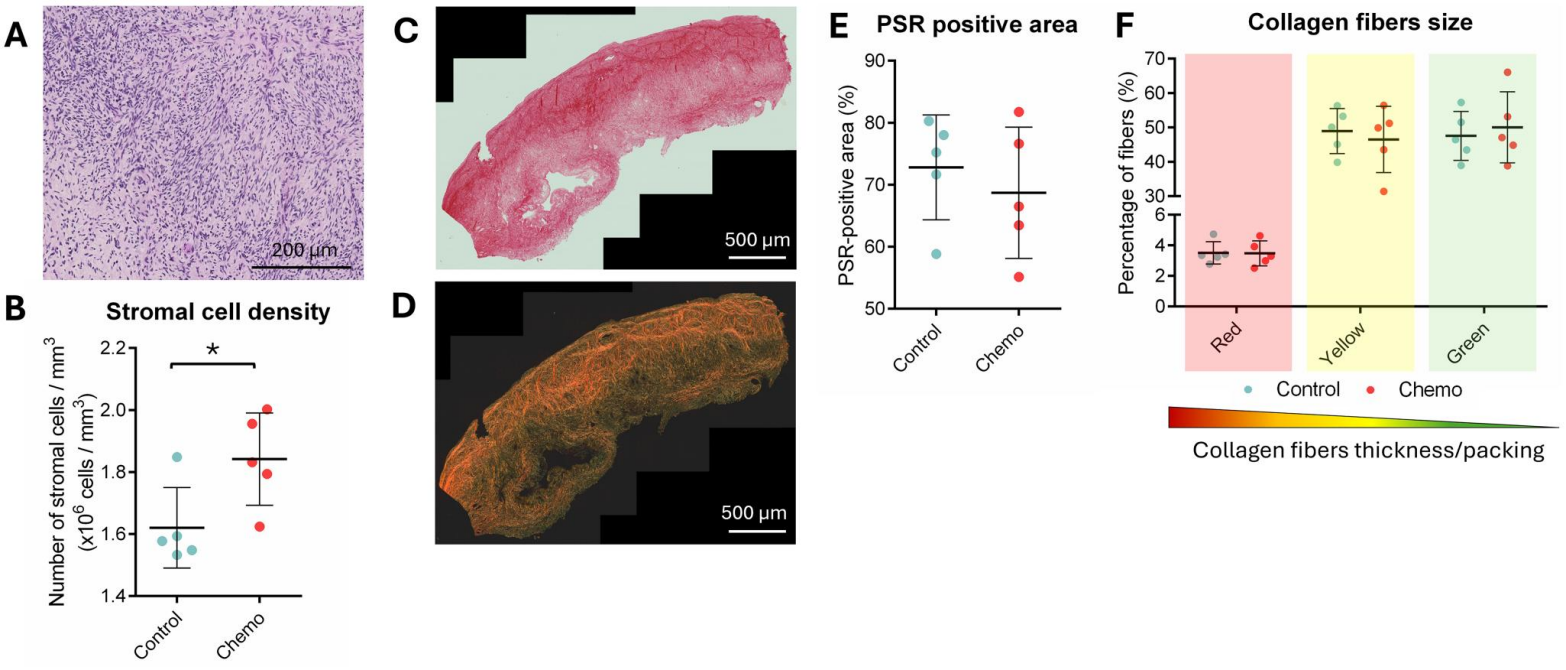
**Extracellular matrix (ECM) remodeling in cryopreserved-thawed ovarian tissue obtained from untreated or chemotherapy-exposed adult women.** (**A**) Representative image of the ovarian stroma. Scale bar = 200 µm. (**B**) Quantification of stromal cell density. (**C**, **D**). Representative images of a human ovarian cortical sections stained with picrosirius red (PSR) and visualized under brightfield (C) or polarized light (D). Scale bar = 500 µm. (**E**) Quantification of the percentage of PicroSirius red (PSR)-positive area. (**F**) Assessment of collagen fibers thickness and packing through the quantification of the relative percentage of each collagen color type as seen under polarized light. Data are expressed as scatter plots ± SD; N = 5 per experimental group. * *P* < 0.05.

Stromal changes including fibrosis have been proposed to contribute to chemotherapy-induced follicle loss (Meirow *et al.*, 2007). Here, stromal cell density was shown to increase following chemotherapy exposure, from 1.62 ± 0.13 × 10^6^ cells/mm^3^ in controls to 1.84 ± 0.15 × 10^6^ cells/mm^3^ in the chemo-treated group (*P* = 0.036; Fig. 4A and B). Fibrosis level, as determined by PSR staining for collagen I and III, was unchanged in ovaries exposed or not to chemotherapy (∼70% PSR-positive area; *P* = 0.518; Fig. 4C and E). Collagen fibers thickness and packing density were further assessed under polarized light (Fig. 4D). Overall, the thickest (red) fibers were the least abundant in the human ovarian cortex and represented ∼3% of total collagen, versus ∼48% and ∼49% for the mid-sized (yellow) and thin (green) fibers, respectively. When comparing the two experimental groups, all fiber types remained stable and similarly distributed (thick fibers, *P* = 0.949; mid-sized fibers, *P* = 0.653; thin fibers, *P* = 0.673; Fig. 4F). The percentage of PSR-positive area and collagen fibers size were also comparable whatever the treatment and the time interval between last chemotherapy exposure and OTC (Supplementary Fig. S2B, D–F). These data suggest an absence of fibrosis in ovaries from women exposed to low gonadotoxic chemotherapy up to 18 months before OTC.

### First-line chemotherapy does not induce ovarian reserve depletion but impairs follicle morphology

**Figure 5.**
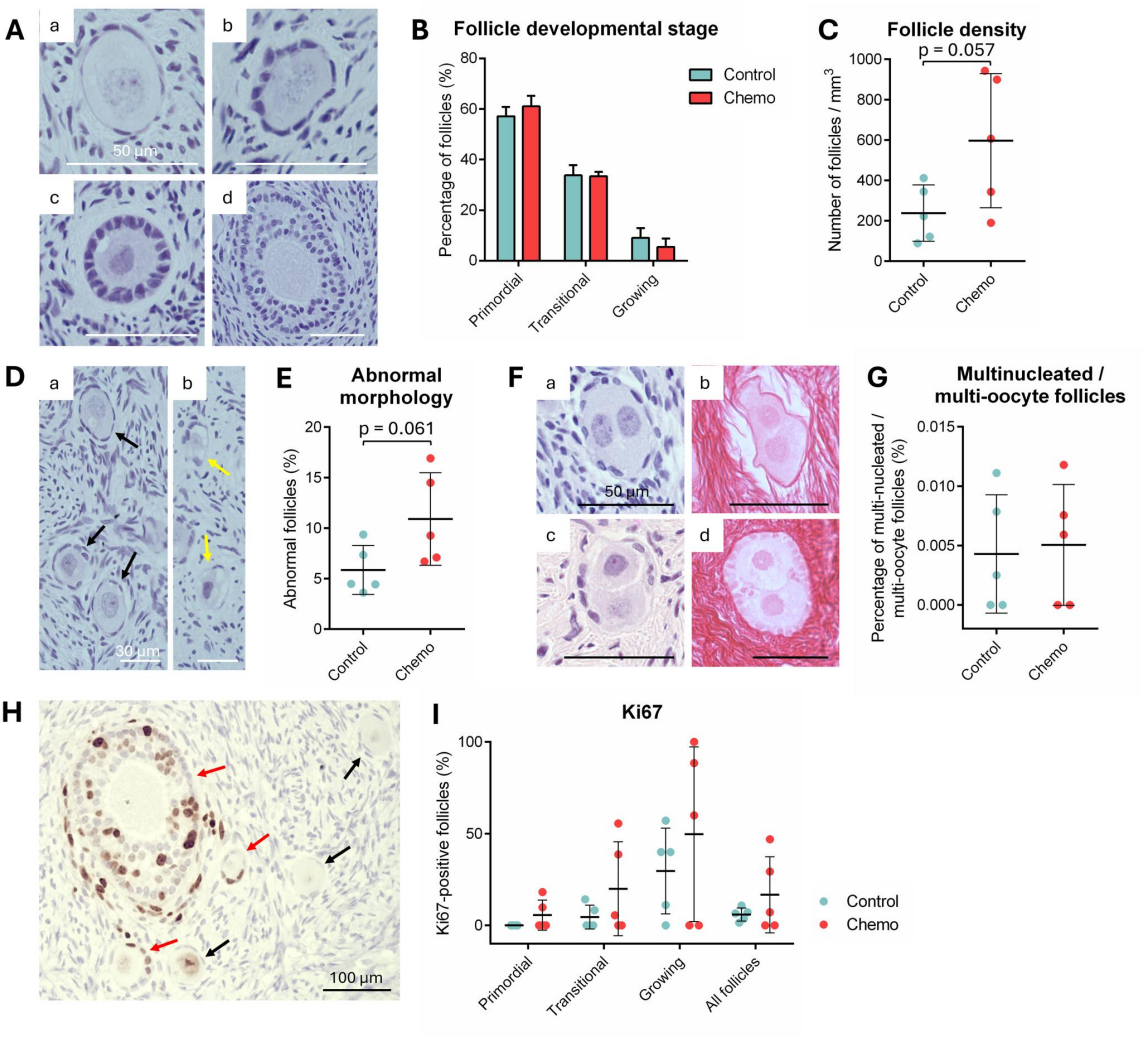
**Follicle characteristics in cryopreserved-thawed ovarian tissue obtained from untreated or chemotherapy-exposed adult women.** (**A**) Photomicrographs of human primordial (a), transitional (b), primary (c), and secondary (d) follicles after hematoxylin and eosin staining. Scale bar = 50 µm. (**B**, **C**) Distribution of follicular stage (as percentage of total, B) and density (C). (**D**) Photomicrograph of follicles displaying a normal (black arrows, a) or abnormal (yellow arrows, b) morphology. Scale bar = 30 µm. (**E**) Quantification of the percentage of morphologically abnormal follicles. (**F**) Photomicrograph of multi-nucleated (a, b) and multi-oocyte (c, d) follicles after hematoxylin and eosin (a, c) and PSR (b, d) staining. Scale bar = 50 µm. (**G**) Quantification of the percentage of multi-nucleated/multi-oocyte follicles in the ovarian tissue. (**H**) Immunohistochemical detection of Ki-67. Red arrows indicate follicles containing Ki-67-positive granulosa cells; black arrows indicate follicles with Ki-67-negative granulosa cells. Scale bar = 100 µm. (**I**) Quantification of the percentage of Ki-67-positive follicles. Data presented as mean ± SD or scatter plots ± SD; N = 5 per experimental group.

To determine the impact of previous chemotherapy exposure on follicular developmental stage, density and morphology, 10 fragments of ovarian tissue (1 per patient) were fixed post-thawing and processed for histological examination. A total of 760 and 2365 follicles were analyzed in the control and chemotherapy groups, respectively. Histological sections of all patients contained primordial follicles, indicating that none of the first-regimen chemotherapy was sterilizing. No difference was detected in the developmental stage of follicles from the control and the chemotherapy groups (primordial follicles, *P* = 0.154; transitional follicles, *P* = 0.847; growing follicles, *P* = 0.158), suggesting no increased follicle activation following chemotherapy exposure (Fig. 5A and B). Follicular density did not decrease but instead tended to be higher in chemotherapy-treated patients compared to age-matched controls (596.68 ± 331.90 follicles/mm^3^ vs. 238.09 ± 139.26 follicles/mm^3^, respectively; *P* = 0.057; Fig. 5C). Analysis of follicle health showed that a higher percentage of follicles from the chemotherapy group displayed an abnormal morphology, including but not limited to misshapen or degenerating oocytes and/or pyknotic GCs (10.9% ± 4.6% vs. 5.9% ± 2.4%; *P* = 0.061; Fig. 5D and E). The presence of multi-oocyte follicles and multi-nucleated oocytes was also recorded (Fig. 5F). Multi-oocyte/multinucleated follicles occurred very rarely in the adult ovary (∼0.005% of all follicles) and their rate was comparable in the two experimental groups (*P* = 0.818; Fig. 5G). Last, we detected follicular Ki-67 as a marker of granulosa cell proliferation (Fig. 5H). No significant changes in the percentage of Ki-67-positive follicles were observed whatever the developmental stage (primordial follicles, *P* = 0.202; transitional follicles, *P* = 0.254; growing follicles, *P* = 0.432; total follicles, *P* = 0.315; N = 828 follicles from 10 patients analyzed) although highly variable (Fig. 5I). Neither the type of chemotherapy regimen nor the time interval between last chemotherapy and OTC modulated Ki-67 expression in granulosa cells (Supplementary Fig. S2K and L).

## Discussion

Compared to the general population, women are 38% less likely to conceive after cancer diagnosis and treatment (Anderson *et al.*, 2018). Although fertility preservation is recommended prior to the initiation of oncological therapies, it is not always feasible due to medical constraints. However, the impact of chemotherapy on the human ovary remains poorly defined, particularly at the microenvironmental level. By combining proteomic profiling with western blotting and (immuno)histological analyses, we generated a comprehensive map of chemotherapy-induced changes in the human ovary, both in the stromal and germ cell compartments. Our data demonstrate that ovaries of women exposed to first-line chemotherapy exhibit signs of immune dysfunction and inflammation, hypoxia, apoptosis, and impaired cell cycle and DNA repair capacity compared to controls. They also highlight that chemotherapy partially disrupts the ECM but does not promote the development of ovarian fibrosis. Last, chemotherapy has a lingering impact on germ cells, with increased follicular apoptosis and DNA damage detectable up to 18 months post-treatment, without a significant reduction in the ovarian reserve.

Our study identified 5209 proteins in the human ovary, a content similar to what has been previously reported (Ouni *et al.*, 2019). Among these, the expression of 239 proteins was significantly altered following chemotherapy exposure, including 162 upregulated and 75 downregulated proteins. GO and pathway enrichment analyses revealed chemotherapy-induced stromal alterations that are involved in immune dysfunction, increased hypoxia and apoptosis, impaired cell cycle and DNA repair capacity and disrupted ECM, findings largely supported at individual protein level. These results align with the dynamic changes observed in other healthy tissues after cancer therapies, including cell apoptosis and senescence, chronic inflammation, ECM remodeling, immune modulation and vascular alterations (Sharma *et al.*, 2014; Stoddart *et al.*, 2020; Haj-Shomaly *et al.*, 2022; Abdul-Rahman *et al.*, 2023). In the ovary, studies analyzing the mouse ovarian transcriptome and secretome following chemotherapy have demonstrated the upregulation of inflammatory/immune responses and cell apoptosis as well as ECM disturbance (Marcozzi *et al.*, 2022; He *et al.*, 2023; Zhou *et al.*, 2024). Imbalance of inflammatory cytokines and infiltration of macrophages and neutrophils has been reported in chemotherapy-treated mice, rats and humans (Fabbri *et al.*, 2019; Deng *et al.*, 2021; Ibrahim *et al.*, 2021; Du *et al.*, 2022). Increased stromal cell senescence and upregulation of cell cycle inhibitors including p53, p21 and p27 have also been described in chemotherapy-exposed mouse ovaries (Dai *et al.*, 2022). Moreover, higher tissue hypoxia is likely a result of vascular damage caused by chemotherapy (Meirow *et al.*, 2007; Soleimani *et al.*, 2011; Bildik *et al.*, 2015; Devos *et al.*, 2023). Together, our findings provide an in-depth analysis of the human ovarian proteome after chemotherapy exposure, identifying conserved dysregulated biological processes across organs and species. PCA analysis also illustrated major proteome differences between the short-interval chemotherapy group and both the long-interval chemotherapy and control groups, suggesting a marked but transient impact of chemotherapy on the ovarian proteome. This observation offers valuable insights into the temporal events of ovarian stromal changes and suggests that first-line chemotherapy-induced microenvironmental damage may be reversible over time.

To gain deeper insight into chemotherapy-induced microenvironmental changes, we combined molecular profiling with histological analyses. Most chemotherapeutic agents exert their cytotoxic effects by inducing DNA double-strand breaks, interfering with the cell cycle and/or generating free radicals that lead to cell cycle arrest and apoptosis (Spears *et al.*, 2019). Although mass spectrometry revealed significant upregulation of apoptosis and downregulation of DNA repair pathways in chemotherapy-treated ovaries, TUNEL assay did not detect differences in stromal apoptosis between the two experimental groups, an observation also reported in chemotherapy-exposed mouse ovaries (Roti Roti *et al.*, 2012). This discrepancy may be attributed to the sensitivity of mass spectrometry in detecting early and intermediate apoptotic events or more subtle molecular changes, whereas TUNEL assay identifies late-stage apoptosis characterized by mid- to extensive DNA fragmentation. Extensive cell death could create a specific microenvironment characterized by high levels of oxidative stress and inflammation, as previously demonstrated in chemotherapy-treated mice (Jiang *et al.*, 2019; Dai *et al.*, 2022; Zhang *et al.*, 2023). In our cohort, oxidative stress levels remained stable between groups. Administration of multiple small doses of chemotherapy compared to one single high dose has similarly been shown to reduce side effects, including lower ROS levels, in mouse ovaries (Athira *et al.*, 2020). Mass spectrometry also indicated cell cycle arrest in chemotherapy-exposed ovaries, yet histological analyses revealed increased stromal cell density post-treatment. We hypothesize that the ovarian stroma undergoes compensatory stromal proliferation in response to chemotherapy-induced injury to restore homeostasis and promote tissue regeneration. Interestingly, excessive stromal proliferation has been linked to fibrosis, a pathological process observed in human ovaries following chemotherapy exposure (Meirow *et al.*, 2007; Pampanini *et al.*, 2019; Shai *et al.*, 2021). Our data suggest that first-line chemotherapy partially disrupts the ovarian ECM, particularly through the upregulation of ECM1 and downregulation of COL1A1 (Supplementary Tables S1 and S2). ECM1 has been shown to have antifibrotic functions in the liver (Link *et al.*, 2025), while COL1A1 is typically associated with fibrosis in the ovary (Umehara *et al.*, 2022; Del Valle *et al.*, 2025). Their imbalance in our study, along with the absence of changes in PSR staining and collagen fiber types, suggest that chemotherapy does not lead to the establishment of local fibrosis, even after a prolonged interval between chemotherapy and OTC. These findings differ from some earlier reports (Meirow *et al.*, 2007; Pampanini *et al.*, 2019; Shai *et al.*, 2021). Discrepancies may reflect differences in chemotherapy regimens, cumulative doses, the timing between last chemotherapy exposure and OTC, or the histological methods used (Masson trichrome vs. PicroSirius Red staining). Similar absence of overt fibrosis has been reported in recent studies of women treated with first-line chemotherapy (Devos *et al.*, 2023; Houeis *et al.*, 2025), indicating that fibrosis may not be a consistent feature across all clinical settings and patient populations. Future research should determine whether and how these microenvironmental alterations directly or indirectly influence follicle dynamics.

Primordial follicle depletion is a common feature of chemotherapy-induced ovarian damage (Oktem and Oktay, 2007; Meirow *et al.*, 2010). Several mechanisms have been proposed to explain this loss, including follicular apoptosis and accelerated activation. Our analyses confirmed increased DNA damage and apoptosis in follicles following chemotherapy, as previously reported (Soleimani *et al.*, 2011; Titus *et al.*, 2021; Devos *et al.*, 2023; Labrune *et al.*, 2023), without elevation in follicular oxidative stress. They also show that even low doses of AA could negatively impact ovarian follicles, and that follicular damage lingers up to 18 months post-treatment. In contrast, patients exposed to first-line chemotherapy exhibited neither a decline in follicle density nor a significant change in follicle proportions. These data conflict with histological analyses of clinical, in vitro cultured and xenotransplanted human ovarian samples that have shown that chemotherapy, in particular alkylating agents, induces ovarian atrophy and a marked depletion of the primordial follicle pool (Oktem and Oktay, 2007a, 2007b; Bildik *et al.*, 2015; Shai *et al.*, 2021). More recent studies examining ovarian tissue from patients treated with first-line, lower-dose chemotherapy regimens have consistently found no significant reduction in follicle density although some reported higher rate of atretic follicles (Asadi Azarbaijani *et al.*, 2015; McLaughlin *et al.*, 2017; Pampanini *et al.*, 2019; Devos *et al.*, 2023; Labrune *et al.*, 2023; Houeis *et al.*, 2025). Importantly, in mice, chemotherapy has been shown to trigger follicle loss in a dose-dependent manner (Meirow *et al.*, 1999; Petrillo *et al.*, 2011). These discrepancies across the literature are thus likely due to differences in the type and cumulative dose of chemotherapy. Moreover, despite being age-matched, follicle density in the first-line chemotherapy group tended to be higher than in the control group, a pattern also observed in other studies (McLaughlin *et al.*, 2017; Devos *et al.*, 2023; Houeis *et al.*, 2025). The mechanisms underlying this observation remain unclear. Histological comparisons revealed morphological differences, with an increased rate of abnormal follicles; however, there were no changes in the proportion of multi-nucleated or multi-oocyte follicles, consistent with findings by Houeis and colleagues (Houeis *et al.*, 2025). These data (i) confirm that both quiescent and activated/growing follicles are susceptible to apoptosis and DNA damage following chemotherapy and (ii) demonstrate that first-line chemotherapy does not significantly affect follicular density or induce follicle activation at clinically relevant doses.

Limitations of the study include the limited cohort size, which reduces the ability to detect subtle differences between groups. Moreover, the first-line treatments in the chemotherapy group were not homogeneous, with variability in the chemotherapy regimens (agents used, dose, duration) and in the time interval between the last treatment and OTC, which potentially introduces confounding factors. As such, larger cohorts with matched regimen and standardized intervals are needed to confirm the findings. On the other hand, this heterogeneity allowed a preliminary assessment of how various treatment regimens and the interval between the last chemotherapy exposure and OTC affected the ovary (Supplementary Fig. S2). Importantly, these data are exploratory and statistical analyses were not performed given the low N. A larger data set is required to (i) unravel potential differences in gonadotoxicity between specific chemotherapy-regimens and (ii) distinguish between the mechanisms involved in the immediate and delayed phases of chemotherapy-induced ovarian injury.

Overall, our study provides the first detailed description of the human proteome associated with chemotherapy-induced ovarian damage. Our findings underscore the limited yet not negligible gonadotoxic effects of first-line chemotherapy on the ovary and provide evidence that both the stromal and germ cell compartments are damaged. A few retrospective studies have shown that first-line chemotherapy prior to OTC does not adversely affect outcomes after ovarian tissue transplantation, with similar rates of ovarian function restoration, miscarriage, pregnancy and live birth observed between chemotherapy-naive and chemotherapy-exposed patients (Poirot *et al.*, 2019; Shapira *et al.*, 2020; Dolmans *et al.*, 2021). Follow-up studies will be essential to further address the long-term consequences for fertility and reproductive lifespan after transplantation. Our data also emphasize the urgent need for strategies to mitigate these off-target alterations and preserve ovarian function post-treatment. In this respect, our work identifies target proteins that may drive these chemotherapy-induced ovarian damage, offering a foundation for future pharmacoprotection investigations aimed at safeguarding ovarian health during and post cancer treatment.

## Supplementary Material

deaf203_Supplementary_Figure_S1

deaf203_Supplementary_Figure_S2

deaf203_Supplementary_Table_S1

deaf203_Supplementary_Table_S2

## Data Availability

The mass spectrometry proteomics data have been deposited to the ProteomeXchange Consortium via the PRIDE partner repository with the dataset identifier PXD064815.
