# Supplementary material for: Proteomic profiling reveals the molecular signatures of chemotherapy-induced human ovarian damage
Source: Hum Reprod. 2025 Nov 5;40(12):2395–408. doi: 10.1093/humrep/deaf203 (PMC12675412; doi:10.1093/humrep/deaf203)
Supplement: deaf203_Supplementary_Figure_S1 [file deaf203_supplementary_figure_s1.pdf]

OXFORD
human reproduction
Human Reproduction, 2025, 1–1
https://doi.org/10.1093/humrep/deaf203
Supplementary Data

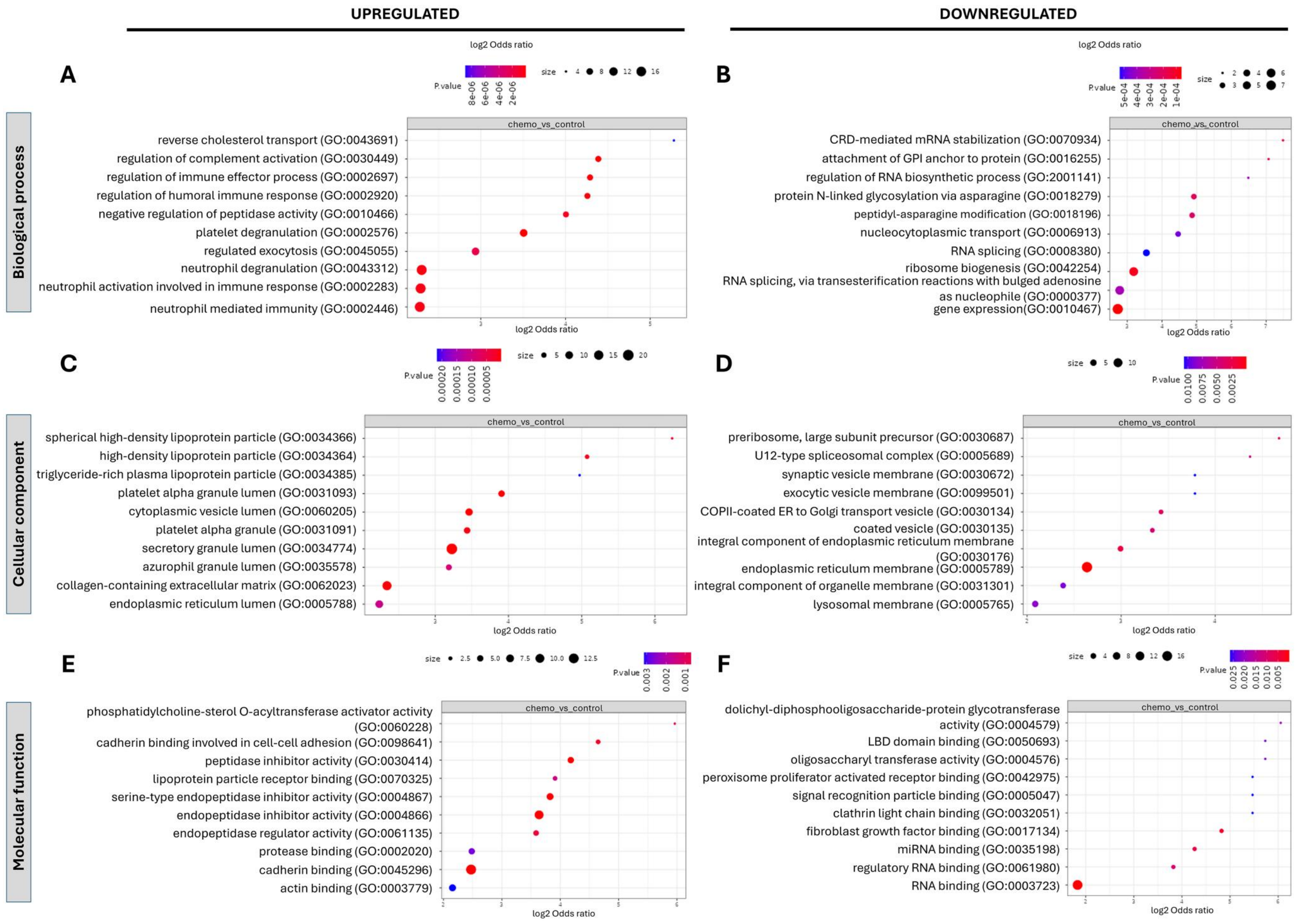

Supplementary Figure S1. Top 10 gene ontology (GO) terms for upregulated or downregulated differentially expressed proteins. The top 10 significantly enriched GO categories of upregulated (**A, C, E**) and downregulated (**B, D, F**) proteins for biological process (A, B), cellular component (C, D) and molecular function (E, F). Only proteins with log2 fold change > 0.585 and *P*-value < 0.05 were considered. For all significant terms the corrected *P*-value, group size and the enrichment factor are displayed. N = 4 in control group, N = 5 in chemotherapy group.
