# Supplementary material for: Proteomic profiling reveals the molecular signatures of chemotherapy-induced human ovarian damage
Source: Hum Reprod. 2025 Nov 5;40(12):2395–408. doi: 10.1093/humrep/deaf203 (PMC12675412; doi:10.1093/humrep/deaf203)
Supplement: deaf203_Supplementary_Figure_S2 [file deaf203_supplementary_figure_s2.pdf]

OXFORD
human reproduction
Human Reproduction, 2025, 1–1
https://doi.org/10.1093/humrep/deaf203
Supplementary Data

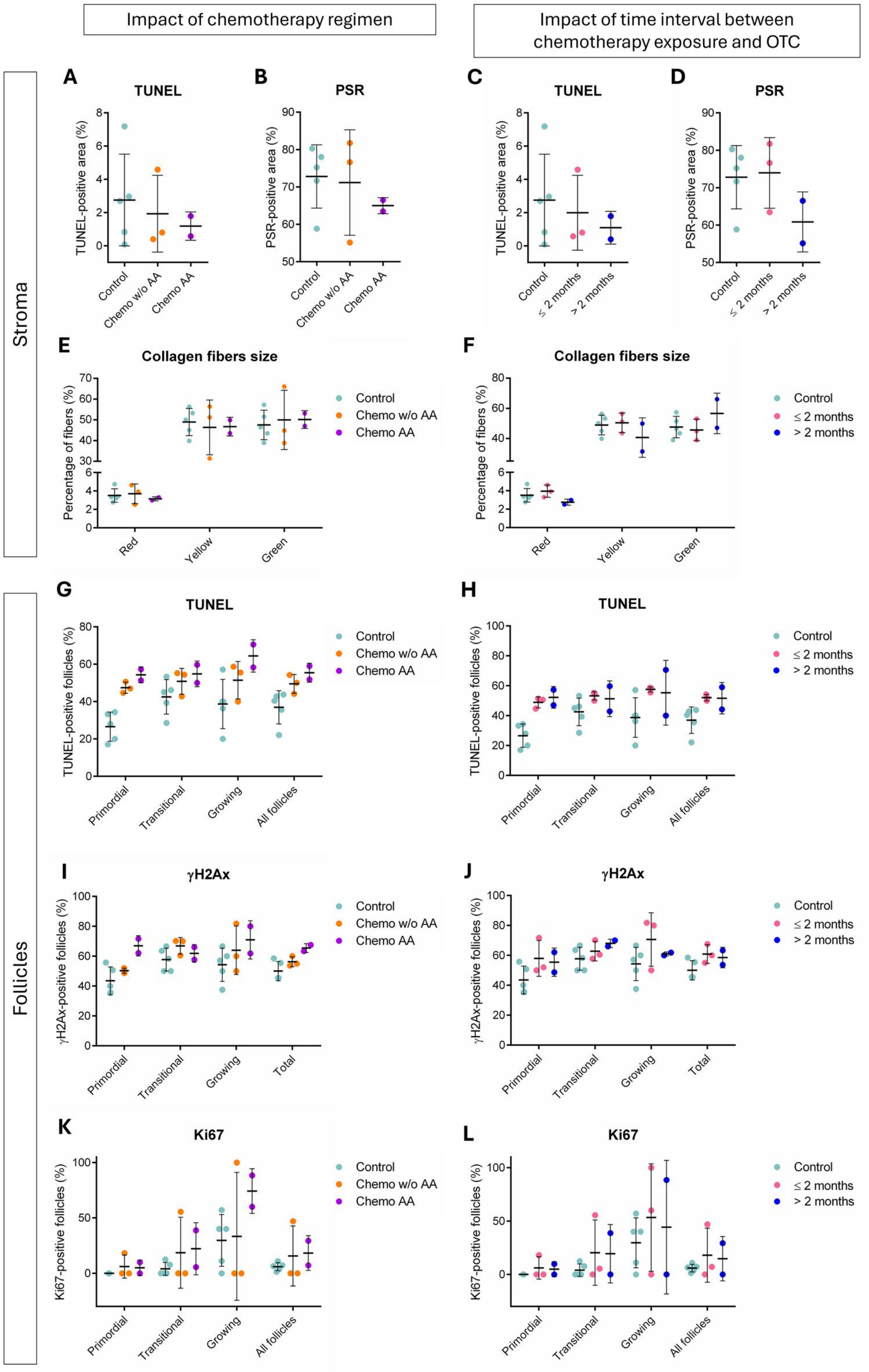

Supplementary Figure S2. Impact of chemotherapy regimen and time interval between last treatment and OTC in the stroma (A-F) and in follicles (G-L). Quantification of TUNEL (A, C, G, H), picrosirius red—PSR (B, D), collagen fibers size (E, F), γH2Ax (I, J) and Ki67 (K, L) according to the chemotherapy regimen (chemotherapy without alkylating agents (AA) vs. chemotherapy with AA; A, B, E, G, I, K) and time interval between last treatment and OTC (≤ 2 months vs. > 2 months; C, D, F, H, J, L). Data presented as scatter plots ± SD; N = 2–5 per experimental group. Chemo w/o AA: chemotherapy without AA; chemo AA: chemotherapy containing AA. No statistical analyses were performed.
