# Supplementary material for: Proteomic profiling reveals the molecular signatures of chemotherapy-induced human ovarian damage
Source: Hum Reprod. 2025 Nov 5;40(12):2395–408. doi: 10.1093/humrep/deaf203 (PMC12675412; doi:10.1093/humrep/deaf203)
Supplement: deaf203_Supplementary_Table_S1 [file deaf203_supplementary_table_s1.pdf]

OXFORD
human reproduction
Human Reproduction, 2025, 1–1
https://doi.org/10.1093/humrep/deaf203
Supplementary Data

Supplementary Table S1. Upregulated proteins in ovarian cortex lysates (chemotherapy vs control).

Protein ID	Gene symbol	Annotation	Adjusted P-value	Log2 (Fold Change)
F6TLX2	GLOD4	glyoxalase domain containing 4	9.93E-05	0.83
P49773	HINT1	histidine triad nucleotide binding protein 1	9.93E-05	0.93
Q04760	GLO1	glyoxalase I	5.74E-04	0.59
P09960	LTA4H	leukotriene A4 hydrolase	5.74E-04	0.61
Q15417	CNN3	calponin 3	5.74E-04	0.76
P58546	MTPN	myotrophin	5.74E-04	0.78
Q8IWE2	FAM114A1	family with sequence similarity 114 member A1	5.74E-04	0.88
Q15126	PMVK	phosphomevalonate kinase	5.74E-04	0.99
P02647	APOA1	apolipoprotein A1	7.71E-04	1.76
Q15181	PPA1	inorganic pyrophosphatase 1	8.09E-04	0.64
P61956	SUMO2	small ubiquitin like modifier 2	8.09E-04	0.82
P05783	KRT18	keratin 18	8.09E-04	1.04
P05155	SERPING1	serpin family G member 1	8.09E-04	1.29
P07741	APRT	adenine phosphoribosyltransferase	8.13E-04	0.74
Q9H6Z4	RANBP3	RAN binding protein 3	9.72E-04	0.92
O95861	BPNT1	3'(2'). 5'-bisphosphate nucleotidase 1	1.12E-03	0.73
P36405	ARL3	ARF like GTPase 3	1.14E-03	0.74
Q969E4	TCEAL3	transcription elongation factor A like 3	1.14E-03	0.83
P07108	DBI	translocator protein	1.14E-03	0.91
P28161	GSTM2	glutathione S-transferase mu 2	1.35E-03	0.91
P02787	TF	transferrin	1.41E-03	1.30
Q8WZA0	LZIC	leucine zipper and CTNNBIP1 domain containing	1.60E-03	1.00
P68036	UBE2L3	ubiquitin conjugating enzyme E2 L3	1.85E-03	0.59
P18669	PGAM1	phosphoglycerate mutase family member 4	1.85E-03	0.63
Q8WW12	PCNP	PEST proteolytic signal containing nuclear protein	1.85E-03	0.68
Q9NVZ3	NECAP2	NECAP endocytosis associated 2	1.85E-03	0.75
Q96IU4	ABHD14B.1	abhydrolase domain containing 14B	1.85E-03	0.94
Q96IJ6	GMPPA	GDP-mannose pyrophosphorylase A	1.85E-03	1.00
P01024	C3	complement C3	1.86E-03	0.95
P02765	AHSG	alpha 2-HS glycoprotein	1.86E-03	1.66
O75347	TBCA	tubulin folding cofactor A	1.90E-03	0.63
Q6XQN6	NAPRT	nicotinate phosphoribosyltransferase	2.01E-03	0.59
P30740	SERPINB1	serpin family B member 1	2.33E-03	0.62
P41236	PPP1R2	protein phosphatase 1 regulatory inhibitor subunit 2	2.33E-03	0.93
B4DUC8	MTAP	methylthioadenosine phosphorylase	2.52E-03	0.61
Q15121	PEA15.1	proliferation and apoptosis adaptor protein 15	2.52E-03	0.67
P14550	AKR1A1	aldo-keto reductase family 1 member A1	2.52E-03	0.74
Q9BRA2	TXNDC17	thioredoxin domain containing 17	2.52E-03	0.75
Q2TAA2	IAH1	isoamyl acetate hydrolyzing esterase 1 (putative)	2.52E-03	0.96
P05413	FABP3	fatty acid binding protein 3	2.52E-03	1.39
Q9UMY4	SNX12	sorting nexin 12	2.58E-03	0.65
A0A0A6YYH1	C15orf38-AP3S2	ARPIN-AP3S2 readthrough	2.58E-03	0.68
Q16610	ECM1	extracellular matrix protein 1	2.58E-03	0.80
Q01469	FABP5	fatty acid binding protein 5	2.59E-03	0.95
Q9NRX4	PHPT1	phosphohistidine phosphatase 1	2.66E-03	0.66
P19823	ITIH2	inter-alpha-trypsin inhibitor heavy chain 2	2.66E-03	1.16
Q9BVG4	PBDC1	polysaccharide biosynthesis domain containing 1	3.06E-03	0.64
P36543	ATP6V1E1	ATPase H+ transporting V1 subunit E1	3.06E-03	0.69
Q96AT9	RPE	ribulose-5-phosphate-3-epimerase	3.06E-03	1.04
P01023	A2M	alpha-2-macroglobulin	3.06E-03	1.56
O14618	CCS	copper chaperone for superoxide dismutase	3.08E-03	0.67
Q9Y2V2	CARHSP1	calcium regulated heat stable protein 1	3.08E-03	0.73
P37802	TAGLN2	transgelin 2	3.08E-03	0.90
O00193	SMAP	chromosome 11 open reading frame 58	3.08E-03	0.97
E9PNP3	AAMDC	adipogenesis associated Mth938 domain containing	3.20E-03	1.17
Q9Y5Z4	HEBP2	heme binding protein 2	3.31E-03	0.72
C9JC84	FGG	fibrinogen gamma chain	3.31E-03	1.26
O14737	PDCD5	programmed cell death 5	3.52E-03	0.68
O60493	SNX3	sorting nexin 3	3.55E-03	0.62
P62328	TMSB4X	thymosin beta 4 X-linked	3.55E-03	0.66
Q08752	PPID	peptidylprolyl isomerase D	3.66E-03	0.84
A0A1W2PRU0	ENSA	endosulfine alpha	3.66E-03	0.85
Q9BS40	LXN	latexin	3.66E-03	0.88

(continued)

OXFORD
human reproduction
Human Reproduction, 2025, 1–1
https://doi.org/10.1093/humrep/deaf203
Supplementary Data

Supplementary Table S1. (continued)

Protein ID	Gene symbol	Annotation	Adjusted P-value	Log2 (Fold Change)
J3KQ18	DDT	D-dopachrome tautomerase like	3.66E-03	0.91
Q9BRF8	CPPED1	calcineurin like phosphoesterase domain containing 1	3.86E-03	0.76
O75368	SH3BGRL	SH3 domain binding glutamate rich protein like	3.97E-03	0.76
P16949	STMN1	stathmin 1	4.08E-03	0.72
P09455	RBP1	AT-rich interaction domain 4A	4.40E-03	0.66
P04264	KRT1	keratin 1	4.40E-03	1.90
O95372	LYPLA2	lysophospholipase 2	4.42E-03	0.90
O95336	PGLS	6-phosphogluconolactonase	4.82E-03	0.75
O00151	PDLIM1	PDZ and LIM domain 1	4.82E-03	1.20
F8W9U3	ABHD14B	abhydrolase domain containing 14B	4.84E-03	0.67
O76070	SNCG	synuclein gamma	4.84E-03	1.22
P00450	CP	ceruloplasmin	4.84E-03	1.49
A0A6Q8PH20	CAST	RNA polymerase I subunit G	4.93E-03	0.68
Q15847	ADIRF	adipogenesis regulatory factor	5.08E-03	1.31
P09104	ENO2	enolase 2	5.46E-03	0.84
O60664	PLIN3	perilipin 3	5.58E-03	0.76
P63313	TMSB10	thymosin beta 10	5.58E-03	0.91
P21266	GSTM3	glutathione S-transferase mu 3	6.09E-03	0.59
Q5SRP5	APOM	apolipoprotein M	6.13E-03	0.71
P01011	SERPINA3	serpin family A member 3	6.44E-03	2.05
P51452	DUSP3	dual specificity phosphatase 3	6.49E-03	0.59
B1AKZ5	PEA15	proliferation and apoptosis adaptor protein 15	6.49E-03	0.63
P46109	CRKL	CRK like proto-oncogene. adaptor protein	6.72E-03	0.59
A0A024QZX5	SERPINB6	serpin family B member 6	6.72E-03	0.65
P30085	CMPK1	cytidine/uridine monophosphate kinase 1	6.72E-03	0.73
P08727	KRT19	keratin 19	6.89E-03	1.14
Q9NX46	ADPRS	ADP-ribosylserine hydrolase	7.30E-03	0.63
P46108	CRK	CRK proto-oncogene. adaptor protein	7.30E-03	0.64
P13807	GYS1	glycogen synthase 1	7.30E-03	0.68
O75223	GGCT	gamma-glutamylcyclotransferase	7.30E-03	0.74
Q9HA64	FN3KRP	fructosamine 3 kinase related protein	7.30E-03	0.81
Q9NZJ9	NUDT4	nudix hydrolase 4	7.30E-03	0.85
Q13541	EIF4EBP1	eukaryotic translation initiation factor 4E binding protein 1	7.30E-03	0.88
Q9UHD9	UBQLN2	ubiquilin 2	7.33E-03	0.66
P00966	ASS1	argininosuccinate synthase 1	7.66E-03	0.59
P02649	APOE	apolipoprotein E	7.66E-03	1.05
Q9Y4E8	USP15	ubiquitin specific peptidase 15	7.75E-03	0.68
P02774	GC	GC vitamin D binding protein	8.04E-03	1.43
P29218	IMPA1	inositol monophosphatase 1	8.88E-03	0.74
Q13228	SELENBP1	selenium binding protein 1	9.43E-03	0.64
G3V1A6	GSDMD	gasdermin D	9.46E-03	1.19
P40855	PEX19	peroxisomal biogenesis factor 19	1.03E-02	0.72
P48960	ADGRE5	adhesion G protein-coupled receptor E5	1.07E-02	1.25
Q9H3G5	CPVL	carboxypeptidase vitellogenic like	1.10E-02	0.73
Q15843	NEDD8	NEDD8 ubiquitin like modifier	1.12E-02	1.01
Q14019	COTL1	coactosin like F-actin binding protein 1	1.14E-02	0.77
Q6GMV3	PTRHD1	peptidyl-tRNA hydrolase domain containing 1	1.17E-02	0.64
O43399	TPD52L2	TPD52 like 2	1.34E-02	0.63
Q16527	CSRP2	cysteine and glycine rich protein 2	1.35E-02	0.86
P52566	ARHGDIB	Rho GDP dissociation inhibitor beta	1.40E-02	1.20
Q01995	TAGLN	transgelin	1.40E-02	1.32
Q53G44	IFI44L	interferon induced protein 44 like	1.41E-02	0.82
O95721	SNAP29	synaptosome associated protein 29	1.41E-02	0.86
P10909	CLU	clusterin	1.41E-02	0.92
Q6UUV7	CRTC3	CREB regulated transcription coactivator 3	1.41E-02	0.94
O14558	HSPB6	heat shock protein family B (small) member 6	1.41E-02	1.09
P15311	EZR.1	ezrin	1.51E-02	1.30
Q9H008	LHPP	phospholysine phosphohistidine inorganic pyrophosphate phosphatase	1.65E-02	0.72
Q92882	OSTF1	osteoclast stimulating factor 1	1.65E-02	0.93
Q9NTX5	ECHDC1	ethylmalonyl-CoA decarboxylase 1	1.86E-02	1.02
P02753	RBP4	retinol binding protein 4	1.86E-02	1.73
P35813	PPM1A	protein phosphatase. Mg2+/Mn2+ dependent 1A	1.87E-02	0.78
Q13158	FADD	Fas associated via death domain	1.87E-02	1.02
Q9H299	SH3BGRL3	SH3 domain binding glutamate rich protein like 3	2.03E-02	0.59

(continued)

Human Reproduction, 2025, 1–1
https://doi.org/10.1093/humrep/deaf203
Supplementary Data

Supplementary Table S1. (continued)

Protein ID	Gene symbol	Annotation	Adjusted P-value	Log2 (Fold Change)
P07205	PGK2	phosphoglycerate kinase 2	2.10E-02	0.63
Q9NUQ8	ABCF3	ATP binding cassette subfamily F member 3	2.11E-02	0.69
Q9BV57	ADI1	acireductone dioxygenase 1	2.16E-02	0.61
P52657	GTF2A2	general transcription factor IIA subunit 2	2.20E-02	0.92
O14497	ARID1A	AT-rich interaction domain 1A	2.31E-02	0.91
P07738	BPGM	bisphosphoglycerate mutase	2.33E-02	0.63
P51570	GALK1	galactokinase 1	2.38E-02	0.63
P00326	ADH1C	alcohol dehydrogenase 1C (class I). gamma polypeptide	2.45E-02	1.84
Q8IYS1	PM20D2	peptidase M20 domain containing 2	2.46E-02	0.66
Q9H425	C1orf198	chromosome 1 open reading frame 198	2.55E-02	0.60
Q96B45	BORCS7	BLOC-1 related complex subunit 7	2.55E-02	0.72
Q8IV38	ANKMY2	ankyrin repeat and MYND domain containing 2	2.60E-02	0.79
Q8NFU3	TSTD1	thiosulfate sulfurtransferase like domain containing 1	2.62E-02	0.91
Q15714	TSC22D1	TSC22 domain family member 1	2.64E-02	0.84
Q96IY4	CPB2	carboxypeptidase B2	2.75E-02	1.05
P50453	SERPINB9	serpin family B member 9	2.85E-02	0.77
Q9Y365	STARD10	StAR related lipid transfer domain containing 10	2.86E-02	0.65
Q9H3Q1	CDC42EP4	CDC42 effector protein 4	2.86E-02	0.77
P49354	FNTA	farnesyltransferase. CAAX box. subunit alpha	3.27E-02	0.70
A0A0G2JPR0	C4A	complement C4A (Chido/Rodgers blood group)	3.27E-02	0.88
P15090	FABP4	fatty acid binding protein 4	3.33E-02	0.80
B9A064	IGLL5	immunoglobulin lambda like polypeptide 5	3.49E-02	0.72
P05156	CFI	complement factor I	3.51E-02	0.99
O75914	PAK3	Rho guanine nucleotide exchange factor 7	3.53E-02	0.81
Q8NHH9	ATL2	atlastin GTPase 2	3.62E-02	0.94
P13647	KRT5	keratin 5	3.62E-02	1.53
P46527	CDKN1B	cyclin dependent kinase inhibitor 1B	3.80E-02	0.88
P19827	ITIH1	inter-alpha-trypsin inhibitor heavy chain 1	3.90E-02	0.87
A0A087WZT3	BOLA2B	bolA family member 2	4.07E-02	0.77
A0A087X232	C1S	complement C1s	4.07E-02	0.84
Q14353	GAMT	guanidinoacetate N-methyltransferase	4.32E-02	1.02
P40121	CAPG	non-SMC condensin I complex subunit G	4.39E-02	0.83
O95456	PSMG1	proteasome assembly chaperone 1	4.82E-02	0.88
P16930	FAH	FA complementation group A	4.92E-02	0.67
