# Supplementary material for: Proteomic profiling reveals the molecular signatures of chemotherapy-induced human ovarian damage
Source: Hum Reprod. 2025 Nov 5;40(12):2395–408. doi: 10.1093/humrep/deaf203 (PMC12675412; doi:10.1093/humrep/deaf203)
Supplement: deaf203_Supplementary_Table_S2 [file deaf203_supplementary_table_s2.pdf]

OXFORD
human reproduction
Human Reproduction, 2025, 1–1
https://doi.org/10.1093/humrep/deaf203
Supplementary Data

Supplementary Table S2. Downregulated proteins in ovarian cortex lysates (chemotherapy vs control).

Protein ID	Gene symbol	Annotation	Adjusted P-value	Log2 (Fold Change)
P04844	RPN2	proteasome 26S subunit. non-ATPase 1	3.11E-03	−0.66
Q9NVV5	AIG1	androgen induced 1	3.55E-03	−0.87
Q96S52	PIGS	phosphatidylinositol glycan anchor biosynthesis class S	4.93E-03	−0.63
Q13445	TMED1	transmembrane p24 trafficking protein 1	5.78E-03	−0.89
P61619	SEC61A1	SEC61 translocon subunit alpha 1	6.00E-03	−1.05
Q6Y288	B3GLCT	beta 3-glucosyltransferase	6.43E-03	−0.71
P13473	LAMP2	lysosomal associated membrane protein 2	6.73E-03	−0.86
Q9BVK2	ALG8	ALG8 alpha-1.3-glucosyltransferase	7.30E-03	−1.22
Q9BU23	LMF2	lipase maturation factor 2	7.30E-03	−0.84
Q9Y3A6	TMED5	transmembrane p24 trafficking protein 5	7.30E-03	−0.65
Q9Y3T9	NOC2L	NOC2 like nucleolar associated transcriptional repressor	7.55E-03	−1.05
Q15648	MED1	methyl-CpG binding domain 4. DNA glycosylase	7.75E-03	−1.23
Q9BUN8	DERL1	derlin 1	7.75E-03	−1.09
Q9NRY6	PLSCR3	TMEM256-PLSCR3 readthrough (NMD candidate)	7.94E-03	−0.74
Q9NP58	ABCB6	ATP binding cassette subfamily B member 6 (LAN blood group)	8.96E-03	−0.65
Q5TCI8	LMNA.1	lamin A/C	9.67E-03	−0.94
Q12769	NUP160	nucleoporin 160	9.67E-03	−0.63
O75251	NDUFS7	NADH:ubiquinone oxidoreductase core subunit S7	1.05E-02	−0.94
Q92922	SMARCC1	SWI/SNF related BAF chromatin remodeling complex subunit C1	1.25E-02	−0.90
P09651	HNRNPA1	heterogeneous nuclear ribonucleoprotein A1	1.28E-02	−0.63
P36406	TRIM23	tripartite motif containing 23	1.41E-02	−0.93
P35052	GPC1	glypican 1	1.41E-02	−0.84
O95399	UTS2	urotensin 2	1.42E-02	−0.60
A0A7I2YQN2	SYNCRI.2	synaptotagmin binding cytoplasmic RNA interacting protein	1.43E-02	−0.78
Q92896	GLG1	golgi glycoprotein 1	1.44E-02	−0.59
O14735	CDIPT	CDP-diacylglycerol–inositol 3-phosphatidyltransferase	1.45E-02	−0.59
A0A1W2PNP0	PIGT	phosphatidylinositol glycan anchor biosynthesis class T	1.51E-02	−0.90
P55287	CDH11	cadherin 11	1.67E-02	−1.08
P46778	RPL21	ribosomal protein L21	1.72E-02	−0.78
P80404	ABAT	4-aminobutyrate aminotransferase	1.72E-02	−0.77
P27338	MAOB	monoamine oxidase B	1.87E-02	−0.65
P61803	DAD1	defender against cell death 1	1.90E-02	−0.92
Q8WVM7	STAG1	prostate transmembrane protein. androgen induced 1	1.90E-02	−0.62
Q96CT7	CCDC124	coiled-coil domain containing 124	2.05E-02	−0.62
Q15910	EZH2	enhancer of zeste 2 polycomb repressive complex 2 subunit	2.08E-02	−0.64
Q9BWU0	SLC4A1AP	solute carrier family 4 member 1 adaptor protein	2.11E-02	−1.68
P55061	TMBIM6	transmembrane BAX inhibitor motif containing 6	2.11E-02	−0.82
Q99805	TM9SF2	transmembrane 9 superfamily member 2	2.17E-02	−1.28
P52298	NCBP2	nuclear cap binding protein subunit 2	2.18E-02	−0.78
Q16563	SYPL1	synaptophysin like 1	2.20E-02	−0.83
P09486	SPARC	secreted protein acidic and cysteine rich	2.20E-02	−0.68
Q8NBJ4	GOLM1	golgi membrane protein 1	2.38E-02	−1.04
P62891	RPL39	ribosomal protein L39	2.55E-02	−0.67
P62316	SNRPD2	small nuclear ribonucleoprotein D2 polypeptide	2.55E-02	−0.62
A0A140T912	HLA-C.1	major histocompatibility complex. class I. C	2.63E-02	−1.01
Q99943	AGPAT1	1-acylglycerol-3-phosphate O-acyltransferase 1	2.63E-02	−0.71
Q9H061	TMEM126A	transmembrane protein 126A	2.66E-02	−0.80
Q9H6K4	OPA3	outer mitochondrial membrane lipid metabolism regulator OPA3	2.70E-02	−0.71
Q96EF6	FBXO17	F-box protein 17	2.81E-02	−0.65
O43759	SYNGR1	synaptogyrin 1	2.86E-02	−0.77
Q9NUD5	ZCCHC3	zinc finger CCHC-type containing 3	2.94E-02	−0.67
P35030	PRSS3	serine protease 3	2.95E-02	−1.32
O43734	TRAF3IP2	TRAF3 interacting protein 2	3.00E-02	−0.94
P00846	MT-ATP6	ATP synthase F0 subunit 6	3.16E-02	−1.07
P17813	ENG	endoglin	3.19E-02	−0.61
Q9BUL9	RPP25	ribonuclease P and MRP subunit p25	3.22E-02	−1.11
O75915	ARL6IP5	ARF like GTPase 6 interacting protein 5	3.22E-02	−0.66
Q9HD45	TM9SF3	transmembrane 9 superfamily member 3	3.22E-02	−0.59
P67809	YBX1	Y-box binding protein 1	3.25E-02	−1.24
P53675	CLTCL1	clathrin heavy chain like 1	3.31E-02	−0.80
O15258	RER1	retention in endoplasmic reticulum sorting receptor 1	3.82E-02	−0.93
Q8IY17	PNPLA6	patatin like phospholipase domain containing 6	3.82E-02	−0.76
Q9UKM9	RALY	RALY heterogeneous nuclear ribonucleoprotein	3.94E-02	−0.69

(continued)

Human Reproduction, 2025, 1–1
https://doi.org/10.1093/humrep/deaf203
Supplementary Data

Supplementary Table S2. (continued)

Protein ID	Gene symbol	Annotation	Adjusted P-value	Log2 (Fold Change)
Q92575	UBXN4	UBX domain protein 4	4.04E-02	−0.68
P02452	COL1A1	collagen type I alpha 1 chain	4.06E-02	−0.67
Q5SY16	NOL9	nucleolar protein 9	4.07E-02	−0.87
Q3SXM5	HSDL1	hydroxysteroid dehydrogenase like 1	4.23E-02	−1.01
A0A7P0TAC5	HTT	solute carrier family 6 member 4	4.23E-02	−0.82
Q9H0S4	DDX47	DEAD-box helicase 47	4.32E-02	−0.77
Q86WA9	SLC26A11	solute carrier family 26 member 11	4.47E-02	−1.75
Q15050	RRS1	ribosome biogenesis regulator 1 homolog	4.52E-02	−0.87
P09001	MRPL3	mitochondrial ribosomal protein L3	4.65E-02	−1.15
Q6P179	ERAP2	endoplasmic reticulum aminopeptidase 2	4.91E-02	−0.71
P54852	EMP3	epithelial membrane protein 3 (MAM blood group)	4.92E-02	−0.78

© The Author(s) 2025. Published by Oxford University Press on behalf of European Society of Human Reproduction and Embryology.
This is an Open Access article distributed under the terms of the Creative Commons Attribution License (https://creativecommons.org/licenses/by/4.0/), which permits unrestricted reuse, distribution, and reproduction in any medium, provided the original work is properly cited.
Human Reproduction, 2025, 00, 1–21
https://doi.org/10.1093/humrep/deaf203
Original Article
